# Collective Perception: A Safety Perspective †

**DOI:** 10.3390/s21010159

**Published:** 2020-12-29

**Authors:** Florian A. Schiegg, Ignacio Llatser, Daniel Bischoff, Georg Volk

**Affiliations:** 1 Corporate Research—Advanced Engineering Connected Mobility Systems, Robert Bosch GmbH, Robert-Bosch-Straße 200, 31139 Hildesheim, Germany; ignacio.llatser@de.bosch.com; 2 Institute of Communications Technology, Leibniz University of Hannover, Appelstraße 9A, 30167 Hannover, Germany; 3 Department of Information and Communication Engineering, Tongji University, 4800 Cao’an Highway (Jiading), Shanghai 201804, China; 4 Active Safety Advanced Technology, Opel Automobile GmbH, Bahnhofsplatz, 65423 Rüsselsheim, Germany; daniel.bischoff@opel-vauxhall.com; 5 Department of Computer Science—Embedded Systems, Eberhard Karls University of Tübingen, Sand 13, 72076 Tübingen, Germany; georg.volk@uni-tuebingen.de

**Keywords:** collective perception, cooperative perception, V2X communication, environmental perception

## Abstract

Vehicle-to-everything (V2X) communication is seen as one of the main enabling technologies for automated vehicles. Collective perception is especially promising, as it allows connected traffic participants to “see through the eyes of others” by sharing sensor-detected objects via V2X communication. Its benefit is typically assessed in terms of the increased object update rate, redundancy, and awareness. To determine the safety improvement thanks to collective perception, the authors introduce new metrics, which quantify the environmental risk awareness of the traffic participants. The performance of the V2X service is then analyzed with the help of the test platform TEPLITS, using real traffic traces from German highways, amounting to over 100 h of total driving time. The results in the considered scenarios clearly show that collective perception not only contributes to the accuracy and integrity of the vehicles’ environmental perception, but also that a V2X market penetration of at least 25% is necessary to increase traffic safety from a “risk of serious traffic accidents” to a “residual hypothetical risk of collisions without minor injuries” for traffic participants equipped with non-redundant 360° sensor systems. These results support the ongoing worldwide standardization efforts of the collective perception service.

## 1. Introduction

One of the main challenges towards highly automated driving is the comprehensive environmental perception of vehicles. Vehicles with a certain degree of driving automation require sufficient knowledge concerning their surroundings to perform safe and comfortable automated maneuvers. State-of-the-art sensor systems, being restricted to line-of-sight detections, often fall short when meeting the strict functional requirements imposed by automated driving applications. V2X communication has emerged as a promising technology to mitigate this lack, allowing traffic participants to share all kinds of information to improve their environmental perception and strengthen their decision-making basis [1].

Examples of V2X applications are cooperative awareness and collective (or cooperative) perception, allowing vehicles to share data about their state and detected objects in the surroundings, respectively. While cooperative awareness has already been standardized, collective perception is in the final stages of standardization in Europe [2] and the US [3]. Collective perception gives traffic participants the possibility to exchange information about objects in their Local Environmental Models (LEMs). The LEM manages object tracks provided by the traffic participants’ object detection sensors, initiating new tracks, merging duplicates and deleting tracks that have not been updated for a given time. Information received using V2X communication is fused into the so-called Global Environmental Model (GEM), which is managed separately from the LEM. The main reasons to maintain two environmental models are (i) to avoid feedback effects by retransmission of received objects, and (ii) to have a fallback system in case the GEM is altered by erroneous or malicious data. The GEM has two main benefits over the LEM: (i) it may contain a larger number of object tracks, e.g., objects which are not detected by sensors because they are located outside the sensor field-of-view but are received via V2X communication, (ii) its object tracks may have a higher data accuracy, e.g., in case that they are measured both via sensor detections and V2X communication, in which case both measurements are fused in order to improve their precision, and (iii) the trust of the managed data is increased due to the higher number of sources.

Currently, two wireless technologies have risen as possible enablers of direct V2X communications. The first one is the Wi-Fi-based IEEE 802.11p standard, also known as Dedicated Short-Range Communication (DSRC) in the US and ITS-G5 in Europe. IEEE 802.11p is mature and ready for deployment [4]. The second cellular-based technology, known as C-V2X, has recently been proposed by the 3rd Generation Partnership Project (3GPP) as an alternative to enable direct communication among vehicles using the side link or PC5 interface [5]. The standard features two operation modes: the network-controlled mode 3 and the autonomous mode 4. Both technologies were designed to operate in the 5.9 GHz frequency band, targeting use cases for driver information and warning. A final decision on which technology should shape the future of V2X communication has not been taken to date.

Furthermore, two new V2X communication technologies with enhanced performance are currently in development. On the one hand, IEEE 802.11bd represents an evolution of IEEE 802.11p, which introduces more advanced PHY and MAC techniques, intending to increase the MAC throughput and communication range of IEEE 802.11p, as well as to provide a form of vehicle positioning through V2X communications. Moreover, IEEE 802.11bd is designed to be interoperable and backward compatible with IEEE 802.11p, while guaranteeing their coexistence and fair channel access opportunities. On the other hand, 5G New Radio (NR) V2X is the successor of C-V2X, including modifications in its radio resource management mechanism and new addressing methods such as unicast and multicast. 

In our previous work, we developed analytical models to evaluate the performance of the collective perception service in IEEE 802.11p [6] and C-V2C mode 4 [7] networks in terms of its impact on the traffic participant’s gained environmental perception. This paper follows a similar approach to analyze the benefit of collective perception based on simulation. The main contributions of this work are threefold: (i) we present a short survey of network- and perception-related evaluation metrics applied to collective perception in the literature, (ii) we define new metrics that quantify the quality of a vehicle environmental model from a more safety-oriented perspective, and (iii) we present simulation results that show the improvement in the vehicle environmental model due to collective perception based on the presented network-, perception-, and safety-related metrics.

## 2. Collective Perception

**Figure 1:**
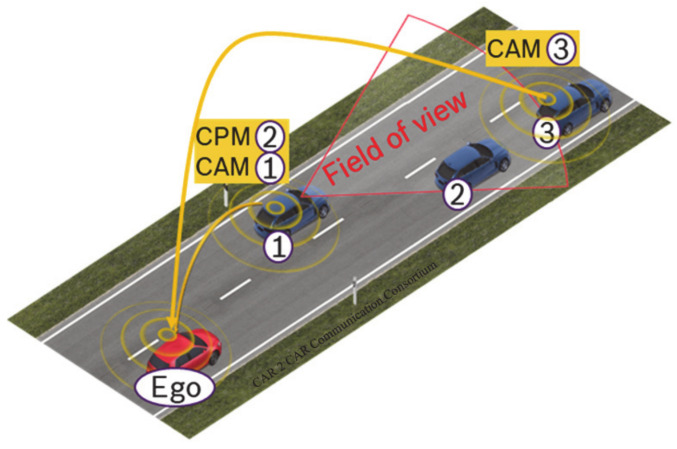
Example of detected vehicles with collective perception. Vehicles 1 and 3 data concerning their state in Cooperative Awareness Messages, while vehicle 1 also transmits vehicle 2′s data in Collective Perception Messages. The ego vehicle can then perceive all other vehicles (also including non-V2X equipped vehicles) [6].

The environmental model of an automated vehicle can be enhanced by the V2X applications of cooperative awareness and collective perception. Cooperative awareness enables vehicles to transmit data about their state via V2X communication, such as their current position, speed, and heading (see Figure 1 vehicles 1 and 3). This service has been standardized worldwide, e.g., with the Cooperative Awareness Message (CAM [8]) in Europe and the Basic Safety Message (BSM [9]) in the US. All V2X-equipped vehicles transmit these messages continuously with a variable frequency between 1 and 10 Hz, depending on the variation of their dynamic state (i.e., current heading, position, and speed) and the measured channel load.

Collective perception [10] allows stations (traffic participants and infrastructure) to inform connected stations of objects (such as pedestrians, obstacles, and other vehicles) detected by their object-tracking sensors (see vehicle 1 in Figure 1). This enables receiving vehicles to extend their environmental model beyond their own sensors’ range. The object data are exchanged through Collective Perception Messages (CPM), currently in standardization by the ETSI [2] to ensure its interoperability among all equipped vehicles.

**Figure 2:**
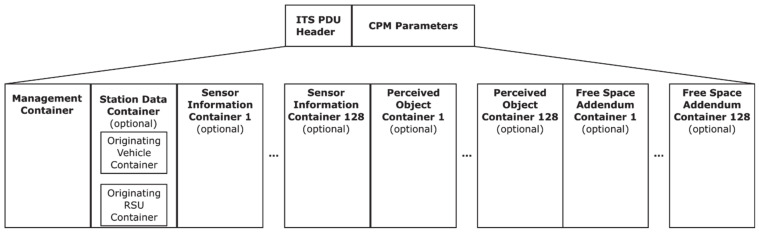
Data structure of a collective perception message [2].

Figure 2 shows the basic structure of a CPM [2], which consists of an ITS protocol data unit header (common to all standardized messages by ETSI) and the actual CPM. The station data and the CPM Management container provide information about the sending station, such as its position and heading. The sensor information container includes details about the on-board object-detection sensors in the sending station, such as their number, range, and aperture angles. The perceived objects’ container is a list of the relevant objects sensed by the sending station, including their relative position and speed, dimensions, and other data. Finally, other containers, such as the free space addendum, contain free space areas detected by the transmitter whenever they cannot be computed by a potential receiver based on the remaining containers.

Another important aspect is the transmission rate of CPMs. Since the frequency band dedicated to V2X communication is limited, it is important to appropriately adapt the generation rate and the included objects in the CPMs. The adaption of the generation rate aims at achieving a good trade-off between the improvement of the environmental model and the additional channel load, thereby preventing channel congestion leading to a decreased performance of V2X communication [10].

The CPM generation rules currently discussed at ETSI are based on the detected objects’ dynamic properties [11,12]. If the object’s dynamic state has changed in a way that would trigger the generation of a CAM by this object, it is then included in the next CPM. Highly dynamic objects are therefore included more often in transmitted CPMs than slow or static objects. The sensor information container is included in the CPM with a fixed frequency of 1 Hz.

Concept and functional benefit of collective perception have been studied with the Artery simulation framework [10,13,14], based on the network simulator OMNeT++. These works found that the vehicle awareness ratio, i.e., how many vehicles it can see around itself, increases significantly when vehicles exchange CPMs in addition to CAMs. Decentralized Congestion Control (DCC) has been found to reduce the vehicle awareness ratio, especially when CAM and CPM are transmitted in the same channel with an increasing V2X-equipment rate [13,14]. However, the impact of the vehicle awareness ratio on the vehicle capability to correctly assess its collision risk based on its environmental model has not been assessed to date. Collective perception is investigated in several European collaborative research projects, such as TransAID [15], AutoNet2030 [16], IMAGinE [17] and MAVEN [18].

## 3. Testing Environment

**Figure 3:**
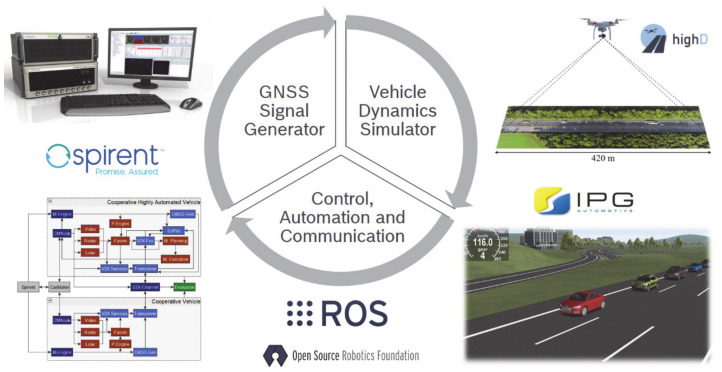
Overview of the main components of the simulation and testing platform TEPLITS [19,20].

Within this work, the TEPLITS platform [19,20] is used to investigate collective perception. TEPLITS was developed for the design, implementation, and testing of new intelligent transportation systems and components. It consists of several modules: the core is the robotics middleware robot operating system (ROS) that orchestrates the interplay of the different components. The platform’s main aspects are presented below, starting with the traffic and vehicle dynamics simulation in Section 3.1 and proceeding with the vehicle sensors in Section 3.2, before finally, the vehicle perception and control is described in Section 3.3. For a more detailed description of TEPLITS, the reader is kindly forwarded to [19,20]. A schematic overview of the main components is offered in Figure 3.

### 3.1. Traffic and Vehicle Dynamics

The accurate modeling of the traffic both on a microscopic and a macroscopic level is essential for analyzing intelligent transportation systems. TEPLITS offers five distinct options for the generation of the trajectories: (i) the online evaluation with real test vehicles [17], (ii) inputting prerecorded object tracks, e.g., from the open source highD [21] and the KoPER datasets [22], (iii) real-track-based data extended, e.g., by Bayesian networks [23], (iv) vehicle tracks generated by the vehicle dynamics simulator CarMaker developed by IPG-Automotive, and (v) artificially generated tracks, such as the spider web scenario [2]. Each of the options has its advantages and disadvantages compared to the others. For this reason, a different option or a combination of these may be chosen depending on the respective investigation needs. For instance, if a more theoretical analysis is targeted, an artificial scenario with precise speeds and routes may be chosen. For a realistic, large-scale scenario investigation, recorded vehicle tracks are preferable. In cases where rarely occurring critical situations are of interest, the tracks may be generated using, e.g., Bayesian networks to multiply these situations. For tests where control of the traffic participants is required, they may be driven by CarMaker, which was extended by a ROS interface in the scope of the public funded project IMAGinE [17]. Further, a combination of real or Bayesian network-generated vehicle tracks with CarMaker is also possible. Should the systems be ready for testing in real test vehicles, a transfer of the modules to the vehicles is made possible due to the common interface of CarMaker and the vehicles.

### 3.2. Vehicle Sensors

Traffic participants in TEPLITS can be equipped with several sensors. These can be divided into three classes:

- Positioning sensors: The main technologies to estimate the ego-position of a traffic participant are satellite navigation, odometry, IMUs, and SLAM. While IMU and odometry are implemented in a simple way in TEPLITS, the state-of-the-art absolute positioning sensor, that is GNSS, is modeled in great detail. For this purpose, the GNSS signal generator SPIRENT GSS7000 developed by Spirent Communications is used. It allows to control a wide range of parameters, such as tropospheric and ionospheric errors, satellite clock and ephemeris errors, obscuration and multipath, receiver antenna characteristics, and RF interference for any given point in time and space. It further allows multi-constellation operation, including GPS, GLONASS, Beidou, and Galileo signals. SLAM has not been implemented in TEPLITS thus far.
- Object tracking sensors: Object tracking sensors, such as radars, LIDARs, and cameras are implemented in CarMaker and the data are outputted over the integrated ROS interfaces. For partially automated vehicles, the human driver must also be considered as an additional or even the primary sensor, releasing driving functions, such as the merging onto a highway. For this reason, the human driver can also be modeled as a sensor in CarMaker [19]. However, it must be noted that the data are processed separately, and not merged into the vehicle’s environmental model. All these object-tracking sensors have also been implemented in ROS based on Bosch internal sensor models for the CarMaker-independent operation of TEPLITS.
- V2X communication: TEPLITS offers two different ways to model the V2X communication: (i) a full stack implementation, and (ii) the application of analytical models developed by the Universidad Miguel Hernandez in Elche, Spain. Generally, the latter is preferable if the focus does not lie on an in-depth analysis of the communication performance, as the full stack software implementation, combined with all the other simulation components, tends to considerably slow down the simulation. The analytical models for 802.11p [24] and C-V2X autonomous mode [25] have been validated with state-of-the-art network simulators and are suitable for most applications.

### 3.3. Vehicle Perception and Control

The information gathered by the vehicle sensors is subsequently processed with the open source robotics middleware platform ROS. The objects are extracted from the measurements, associated and fused into the LEM. The track manager entity is responsible for updating the existing track, creating newly detected ones, and deleting outdated tracked objects. A situation analysis will assess the current traffic situation based on the sensor data and possible further information, e.g., from map services. V2X messages are generated based on these two modules and handed over to the communication control unit for transmission. Received V2X messages are processed and utilized for the generation of GEM and V2X-enhanced situation analysis. Further V2X services, such as collaborative positioning, have also been implemented in TEPLITS and support the generation of a precise decision basis, as it is the GEM and the situation analysis. They form the basis for the maneuver planning of the vehicle. The functions implemented range from an intersection assistant to platooning and from a cooperative Adaptive Cruise Control (c-ACC) to a highway on-ramping assistant, depending on the vehicle’s level of automation. Finally, if CarMaker or a real test vehicle is connected, the maneuver can be executed according to the control commands generated in the maneuver execution module. 

## 4. Evaluation Metrics

In order to quantify the benefit of collective perception several metrics have been proposed in the past. Generally, these can be divided into three groups: (i) those assessing the generated load on the V2X communication network, (ii) those evaluating the benefit of the service in terms of enhanced environmental perception, and (iii) those quantifying the improvements in terms of safety, efficiency, and comfort. Some of the metrics belonging to each of these groups are discussed in the following, citing related work making use of them to evaluate the performance of collective performance.

### 4.1. Network-Related Metrics

Some of the metrics addressing the “costs” of the collective perception service, namely the additionally generated load on the wireless communication channel, are message size, channel busy ratio (CBR), and packet delivery ratio (PDR). As opposed to the metrics quantifying the benefit of collective perception, these metrics are common to all V2X services and thus also used, e.g., to evaluate the performance of CAMs [26] and maneuver coordination messages (MCMs) [27]. As they are well known to the collective perception community, they are described only briefly.

#### 4.1.1. Message Size and Frequency

Two of most straightforward metrics concerning channel resources’ demand by a V2X service are size and frequency of the disseminated messages. New CPM formats have been proposed, effectively reducing the message size, and only slightly compromising the benefit of the service [28,29]. In the same way, other works have introduced new message dissemination rules with positive effects on the message’s transmission frequency and the number of shared objects. Most of these dissemination rules use both message size and frequency to evaluate the effect of the collective perception service on the communication channel [11,12,30,31,32,33,34,35].

#### 4.1.2. Channel Busy Ratio (CBR)

The CBR is a common metric to evaluate the load caused by communicating V2X stations and represents the fraction of time the channel is perceived as busy. As it depends on the previously mentioned message sizes and frequencies, it is a much more direct measure of the “costs” of the collective perception service. The CBR has been employed by several groups to compare CPM dissemination rules in terms of channel utilization [10,11,14,32,34,36].

#### 4.1.3. Packet Delivery Ratio (PDR)

A third state-of-the-art metric computing the effect of the message dissemination on the communication channel is the PDR. It represents the probability of receiving a certain packet, typically in dependence of the CBR and the distance between the transmitting and receiving station. The better the PDR is for a certain set of dissemination rules, the lower the negative impact on the communication channel. This metric and its reciprocal, the packet error rate, have been used among others in [12,32,33,34,35,36,37,38].

### 4.2. Perception-Related Metrics

In contrast to the above-described communication metrics quantifying the “costs” of the collective perception service, the environmental perception metrics measure its “benefit”. While sometimes they are used in combination with communication metrics, they may as well be employed in a stand-alone fashion, e.g., to optimize the benefit of the service with given channel constraints (single vs. multi-channel, dedicated vs. shared channel, etc.). 

#### 4.2.1. Environmental Awareness Ratio (EAR)

Several metrics describing the fraction of environmental elements known to a station (e.g., traffic participant or infrastructure) within a determined region in space and time are collectively referred to as environmental awareness ratio (EAR). The most common form of the EAR is the object awareness ratio (OAR), describing the ratio of objects (generally other traffic participants) perceived by a station. It is typically used for a certain relevant region around the vehicle and for the present time [6,7,10,11,12,14,30,31,32,33,35,39]. In addition, relevant areas further away from the station in question, such as the on-ramping region on a highway, and times in the future, such as the time the on-ramping maneuver is carried out, have been used [19]. In contrast to the OAR, the space awareness ratio (SAR) gives the fraction of the space known to the station within the defined relevance region at a given time. The mentioned on-ramping scenario is a good example where the SAR may be more meaningful than the OAR, since the vehicle is interested in finding a suitable gap between the vehicles in the highway, and only knowing the position of some of the vehicles would not be sufficient. A SAR-like metric is made use of in [19,40,41].

#### 4.2.2. Detected Object Redundancy (DOR), Update Period, and Age of Information (AOI)

Besides the ratio of relevant objects perceived by the station, the redundancy with which these objects are perceived is of interest. Apart from the message generation rules, the DOR is typically caused by a higher number of independent sources, which contributes to the integrity of the data managed in the GEM. A higher DOR generally decreases the GEM update period, i.e., the time between consecutively received measurements of an object in the GEM. The information freshness of the object can be further expressed by its AOI. It is composed of all arising latencies, the major ones being the just introduced update period, the sensor update rate, processing times and further communication delays [7]. Several papers have made use of the DOR [7,12,30,31,40,41,42], the update rate or period [6,7,11,12,32,35], the distance covered by the object between updates [6,7,32], and the AOI [6,7].

#### 4.2.3. Object Tracking Accuracy (OTA)

While EAR and DOR already describe the probability of an object being perceived by a station and the expected number of measurements, detection and tracking accuracies describe how precisely the object state is known. This may be expressed by the deviation of its estimated position with respect to ground truth (e.g., horizontal positioning accuracy), by the errors concerning momenta of higher order, or by the respective covariance matrices. The metric requires more sophisticated processing of the data in the investigated station, i.e., sensor data fusion algorithms, which explains its significantly less frequent use to evaluate collective perception [28,29,37] as compared to, e.g., collaborative positioning [43]. An interesting variation of the OTA is the information entropy, used in [37] to select objects for transmission based on their information value.

### 4.3. Safety, Efficiency, and Comfort Metrics

These metrics are placed at a somewhat higher aggregation level and measure the impact of the vehicle environmental perception on driving safety, efficiency, and comfort. Thus, they are more suitable to describe the overall performance of the collective perception service. Due to the lack of appropriate safety metrics in the literature concerning collective perception, the new environmental risk awareness (ERA) metric is presented in the following and the comprehensive safety metric (CSM) [44] is adapted to suit the evaluation of collective perception. 

#### 4.3.1. Environmental Risk Awareness (ERA)

Given the lack of a simple risk assessment metric for CP, we introduce a new metric that aims at estimating how aware a station is of the risk the current traffic situation bears for it. For this purpose, we propose to compare the perceived risk with the real risk of the environment. The real risk of a situation can be approximated by computing the instantaneous approaching time $t_{i}$ of each relevant traffic object i towards the station. Traffic objects with which a collision is not possible (e.g., driving in the opposite direction of a motorway) are naturally not considered. The inverse of $t_{i}$ is referred to as instantaneous approaching rate and corresponds to the solution of the motion equation:

(1) $$d_{t_{0}}+{\dot{d}}_{t_{0}}t_{i}+\frac{\left({\ddot{d}}_{t_{0}}+{\ddot{d}}_{max}\right)\cdot t_{i}^{2}}{2}=0$$

where $d_{t_{0}}$, ${\dot{d}}_{t_{0}}$ ${\ddot{d}}_{t_{0}}$ are the distance, relative speed, and acceleration of the station and the object in the evaluation timestamp $t_{0}$. The additional parameter ${\ddot{d}}_{max}$ corresponds to the maximal change in the object’s relative dynamics, depending on its current speed and the maximum acceleration, deceleration, and steering angle of the object. The risk posed by this object is then proportional to the approaching rate and the severity of a possible collision $\mu_{i}\;$(e.g., a collision with an object of vastly different dimensions or mass is weighted higher). Thus, the overall risk for the station can be computed from the contributions of all relevant objects with positive approaching times:

(2) $$ERA_{0}=\underset{i;\;t_{i}>0}{\sum }\frac{\mu_{i}}{t_{i}}$$

Similarly, the perceived risk can be computed from the instantaneous approaching rate and the corresponding uncertainties for each dimension of the state vector:

(3) $$ERA_{p}=\underset{i;\;t_{i}>0}{\sum }\frac{\mu_{i}}{t_{i}+\;u\left(t_{i}\right)}$$

where $u\left(t\right)$ corresponds to the uncertainty of the approaching time and can be computed as:

(4) $$u\left(t_{i}\right)=\sqrt{\vec{\nabla }t_{i}{}^{T}\cdot V_{i}\cdot \vec{\nabla }t_{i}}$$

with $V_{i}$ being the covariance matrix for the state of object i. Instead of using a 95% confidence interval, as specified for the CPM [2], higher order protection levels (e.g., 99.99%) may be evaluated depending on the requirements. The station’s environmental risk awareness can now be computed as the perceived risk divided by the real risk:

(5) $$\mathrm{ERA}=\frac{ERA_{p}}{ERA_{0}}$$

The value of ERA ranges from 0 (no perceived risk, leading to poor risk awareness) to 1 (perceived risk equal to real risk, resulting in a perfect risk awareness). Thus, the ERA allows to aggregate all relevant components of the environmental model into one physically meaningful metric, from the EAR to the accuracies of the tracked object states. Furthermore, it weighs objects according to their relevance for the analyzed station, making it a very integer metric.

#### 4.3.2. Comprehensive Safety Metric (CSM)

Ensuring safety is the key component to make sure that autonomous vehicles are ready for their market introduction. For measuring safety, Volk et al. [44] presented a new safety metric that can evaluate the safety of a perception system considering real-time aspects, object relevance, and the possible consequences of undetected objects leading to a potential collision. 

**Figure 4:**
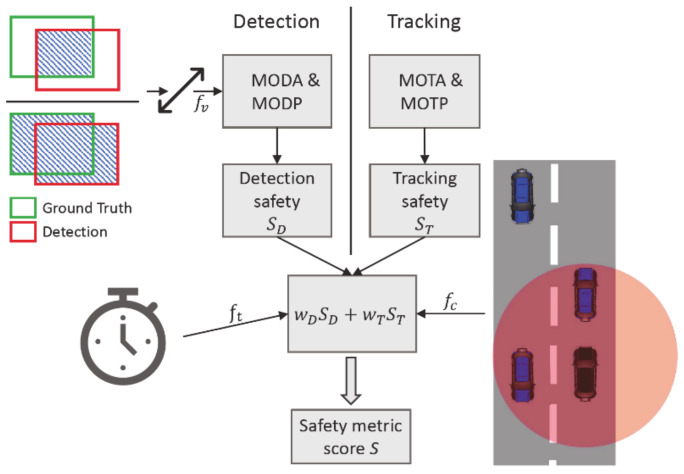
Schematic representation of the Comprehensive Safety Metric as introduced by Volk et al.

Figure 4 gives an overview of the CSM. For its computation tracking and detection quality are evaluated separately, focusing on three main aspects: detection quality, object relevance, and detection times. Closer objects are more safety-relevant than distant ones. Therefore, CSM requires close objects to be detected more accurately. This is achieved by refining the original Intersection over Union (IoU) score with verification factor $f_{v}$ as depicted in Figure 4. This refined IoU is used to calculate the CLEAR metrics Multiple Object Detection Accuracy (MODA), Multiple Object Detection Precision (MODP), Multiple Object Tracking Precision (MOTP), and Multiple Object Tracking Accuracy (MOTA) [45] leading to a distance-based detection quality assessment. Detection and tracking evaluation yield an interim safety result: detection & tracking safety. These are defined as the mean of accuracy and precision scores for each tracking and detection. The interim must be weighted in dependence of the respective use case and are considered to be equally important for the evaluation in this work as proposed by [44]. To incorporate the object’s relevance, CSM uses the Responsibility-Sensitive Safety (RSS) model [46] to define minimal lateral and longitudinal safety distances. If objects are undetected and fall below the defined minimal safety distances, a potential collision will be evaluated. According to the object classes, the severity of a collision is calculated, and the collision factor $f_{c}$ reduces the final safety score S. To incorporate real-time aspects the detection time factor $f_{t}$ is introduced. Longer detection times result in less time for an autonomous system to prevent potential collisions and therefore, reduce the final safety (see also time to plan (TTP) in subsequent sub-subsection). The final safety metric score *S* defined by the CSM as the weighted interim safety result multiplied with the factors $f_{c}$ and $f_{t}$. Overall, it allows for a more precise evaluation compared to standard metrics like average precision as additional safety-relevant factors such as detection times and object relevance are considered.

To apply the metric to collective perception, it had to be slightly modified. Compared to the original CSM [44], undetected objects will be considered with an intersection-over-union score of zero in the MODP. This way true positive detections still get handled the same way, but undetected objects result in an even stricter rating, ensuring that the inclusion of new V2X objects into the GEM improves the safety even if the detection accuracy is low.

#### 4.3.3. Time to Plan (TTP)

Of the three presented metrics within this subsection, the TTP is the most concrete in terms of its functional benefit as it is located at the application layer instead of the facility layer of the V2X protocol stack. In general, it is a measure of the available time for the station to plan a specific maneuver. In other words, the time between the moment at which the station has sufficient information to plan a maneuver until the maneuver is carried out. Günther et al. investigate the time in advance a vehicle learns about an object on the road ahead of it [1]. In another example, Schiegg et al. compute when the vehicle has received sufficient information to determine and head for a gap on the highway [19]. Not surprisingly, together with the change in speed and acceleration, it is the most relevant metric to evaluate the benefits of the MCM in terms of traffic efficiency [47].

## 5. Results and Discussion

The benefit of collective perception is evaluated based on a selection of the metrics presented in the previous chapter. Therefore, a highway scenario was chosen. The two main reasons are (i) the simplicity to describe the scenario, and (ii) the availability of publicly accessible real-world datasets, facilitating comparability and reproducibility. Section 5.1 characterizes the investigated scenarios and simulation parameters before evaluating collective perception in Section 5.2, Section 5.3 and Section 5.4, based on each of the metric categories introduced in the previous chapter. 

### 5.1. Simulation Parameters

The following subsection presents the parameters describing the scenario (Section 5.1.1), the vehicles and their sensors (Section 5.1.2), and the V2X communication (Section 5.1.3).

#### 5.1.1. Scenario

**Figure 5:**
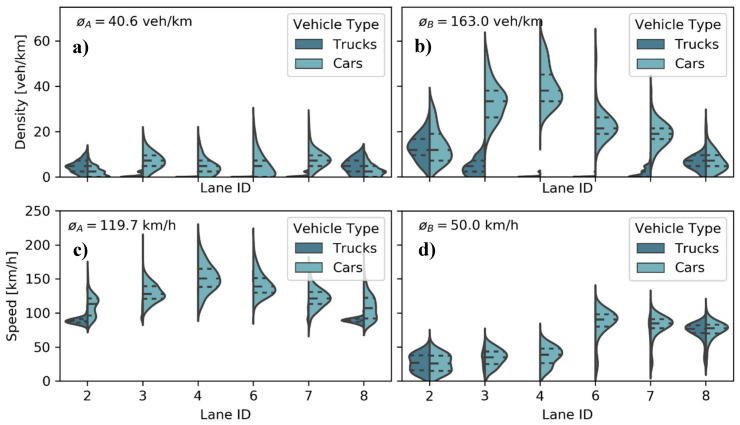
Traffic characterization in terms of: (**a**+**b**) vehicle density and (**c**+**d**) speed distribution for (**a**+**c**) the low and (**b**+**d**) the high traffic density scenario with their respective quartiles (dotted lines). Traffic speed and density have a high impact on the performance of collective perception.

The performance of collective perception strongly depends on the traffic density, the average speed, and the number of lanes [42]. For this reason, two different highway segments with quite different traffic densities were selected in the region of Cologne (see Figure 5 in [21]). The vehicle traces were obtained from the highD dataset [21]. To smoothen the results, up to 10 runs were carried out for the intermediate V2X penetration rates (in this article the terms “market penetration”, “market share” and “V2X equipment rate” are used interchangeably). An overall characterization of both scenarios is given in Table 1. The corresponding vehicle densities and speeds are shown in more detail in Figure 5, differentiating between trucks and vehicles. 

#### 5.1.2. Vehicles and Their Perception

**Figure 6:**
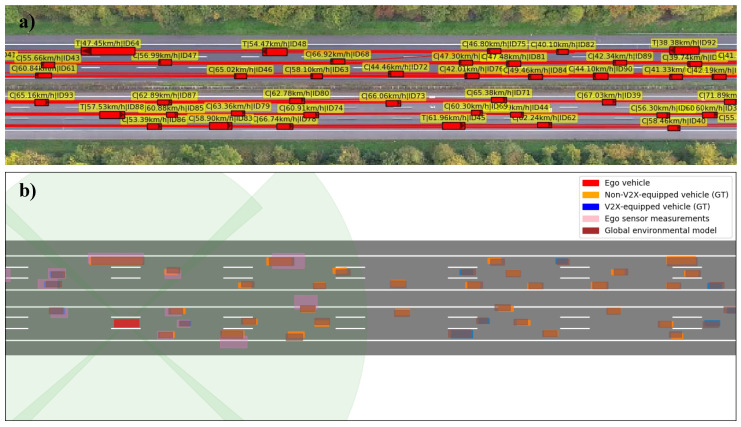
High-density highway segment of scenario B: (**a**) Ground truth positions of the vehicles as contained in the highD dataset; (**b**) Visualization of the environmental perception of a vehicle (red box), showing the sensor measurements (pink boxes) and the fused global environment model (brown boxes) for a V2X market penetration of 25%. They are superposed over the ground truth states’ bounding boxes of the surrounding traffic, differentiating between V2X-equipped (blue boxes) and non-V2X-equipped vehicles (orange boxes).

The high quality of the highD dataset allows to use real vehicle traces and dimensions, with an accuracy of <10 cm (see Figure 6a). For simplicity, the vehicles are all equally equipped with six cameras as depicted in Figure 6b. The camera system has an effective 360° field-of-view and a tracking range of 85 m (shaded green area). Both detection probability and detection accuracy are inversely proportional to the distance of the tracked object and directly proportional to the fraction of its visible cross section. The ego vehicle’s sensor measurements are illustrated in pink. It is clearly visible how the pink bounding-boxes grow with increasing distance from the ego vehicle and some vehicles are not even detected due to an obstructed line-of-sight. It can further be seen that the GEM (brown boxes) reaches far beyond the field-of-view of the ego vehicle’s sensor system includes the shadowed vehicles, and has a higher precision than the stand-alone measurements, clearly demonstrating the benefit of collective perception with the shown 25% market penetration. Both LEM and GEM management facility track objects after their last detection, predicting their state until a given accuracy threshold is exceeded. For the purposes of this work, the threshold is set to lane accuracy as required by the association algorithms in dense scenarios.

#### 5.1.3. V2X Communication

For the evaluation, IEEE 802.11p is used with a QPSK modulation and a coding rate of 0.7. Antenna and average environmental heights, sensing power threshold, background noise and the transmission power were adopted from the analytical model in [6]. The transmitted CPMs correspond to the latest state of standardization at the ETSI [2], following the specified dynamic generation rules. According to practical experience [17] and to foster reproducibility, all optional data fields are filled. For the purposes of this study, ETSI DCC [48] and the redundancy mitigation rules proposed in the technical report [2] are turned off.

### 5.2. Effect on the Communication Channel

The following three subsections discuss the generation of the CPM and its effect on the communication channel. The metrics introduced in Section 4.1 have been analyzed thoroughly in the cited literature and are thus discussed only briefly in the following.

#### 5.2.1. Message Size and Frequency

**Figure 7:**
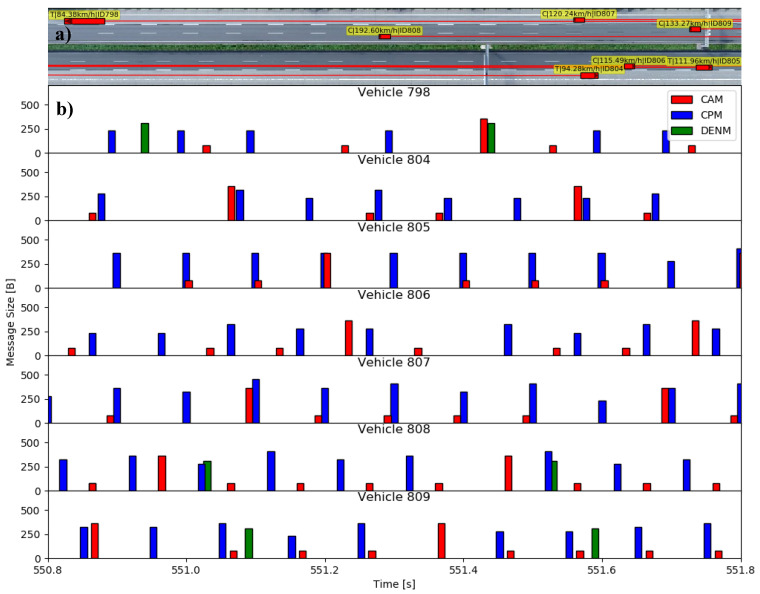
Message generation of the ETSI stack for the low-density scenario: (**a**) Vehicles on a highway section; (**b**) Corresponding generated messages over time. The temporal extension of the messages is only for visualization purposes.

Figure 7 shows a region of scenario A (low density) and the corresponding messages generated on the facility layer of the V2X protocol stack with their respective sizes in bytes. The already standardized CAMs and Decentralized Environmental Notification Messages (DENMs [49]) are shown for comparison purposes but they are not considered for the later evaluation of the collective perception service.

The CAM is of interest for the analysis of the CPM due to their related generation rules. The main dissemination parameter on a highway scenario is the vehicle speed. As can be seen, vehicles driving at higher speeds will have higher CAM generation rates. Vehicle 808 for instance, driving at a speed of ∼193 km/h, generates CAMs every 100 ms, while truck 798, driving at ∼84 km/h, generates a CAM at roughly half of that rate. Every 500 ms a low frequency container with supplementary information is added to the CAM, leading to a significant increase of its size.

While the CAM is generated depending on the transmitting vehicle’s speed, the CPM includes detected objects depending on their measured speed. Since a CPM is generated whenever at least one detected object has been selected for transmission, vehicles disseminate more CPMs than CAMs even at these low traffic densities. Every 1000 ms a sensor container (see Section 2) is added to the CPM.

A timeframe was chosen in which, apart from the CAMs and the CPMs, DENMs are also present. At this moment, the truck (vehicle 798) detects a potentially hazardous situation and transmits a DENM to warn the surrounding vehicles. The DENM is received and immediately retransmitted by vehicle 808. The new DENM is, in turn, received by vehicles 807 and 809, but only retransmitted by the latter, as this vehicle is located further away from the last transmitter (vehicle 808). The DENM dissemination rules allow for further DENMs later in time to warn about the persistence of the event causing them. In this case the DENM is retransmitted by the truck after 500 ms.

Overall, it is evident that the CPM demands significantly more resources compared to CAMs or DENMs, even at this low traffic density. This increases the relevance of channel-aware generation rules. Furthermore, it is much less predictable in both size and periodicity, posing not yet solved problems for the semi-persistent scheduling of C-V2X mode 4 [26].

**Figure 8:**
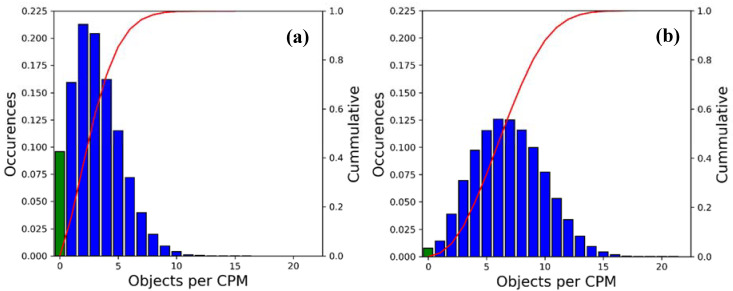
Probability Density Function (PDF; blue bars) and Cumulative Distribution Function (CDF; red line) of the number of objects per CPM for (**a**) the low traffic scenario and (**b**) the high traffic scenario. Not transmitted CPMs are depicted by green bars.

The high variability of the CPM size is represented in more detail in Figure 8. The blue bars show the probability distribution of the number of objects included in a CPM for (a) scenario A and (b) scenario B. The red line shows the cumulative distribution function. As can be seen, the higher vehicular density also increases the number of detected objects, incrementing the average number of included objects per CPM from 2.5 to 6.5. Furthermore, the number of CPMs discarded in lack of perceived objects passing the service’s object inclusion filters (shown by the green bars) decreases with higher density. These findings are in good agreement with [42], as the number of objects in line-of-sight increases faster with rising traffic density than the decrease of their dynamics. However, scenario B is already close to the limit traffic density (20–40 Veh/km/lane depending on the sensor system’s range [42]) where occlusion effects by the dense traffic and the falling object inclusion rate due to the reduced mobility become dominant.

#### 5.2.2. Channel Busy Ratio

**Figure 9:**
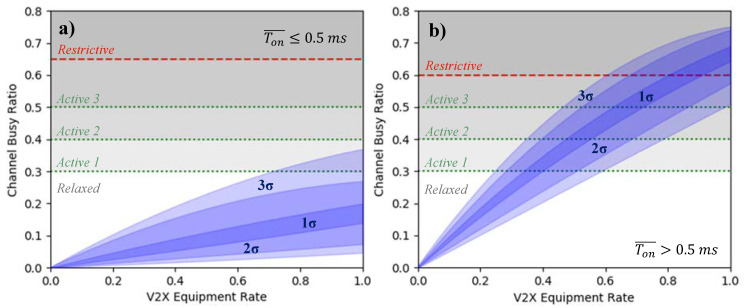
Channel busy ratio for (**a**) the low-density scenario and (**b**) the high-density scenario providing the 1, 2, and 3-standard deviation intervals. The figure further shows the DCC levels of the 5-state ETSI DCC for message durations (**a**) $\bar{T_{on}}\leq 0.5\;\mathrm{ms}$ and (**b**) $\bar{T_{on}}>0.5\;\mathrm{ms}$ [48].

The combined effect of a high number of transmitting stations and the larger average message size can be quantified by the channel busy ratio, representing the fraction of time the channel is perceived as busy by a station, averaged over all V2X-equipped stations. As shown by Figure 9, the channel load in scenario A is very low and remains within the unrestricted reactive DCC state according to the standard [48] for most of the time, even with a full V2X market penetration. Only in rare cases, i.e., a three standard deviation interval, the channel load induces the first active DCC state. However, since the average packet duration is $\bar{T_{on}}\leq 0.5\;\mathrm{ms}$ this translates into a minimum idle time $T_{off}\geq 100\;\mathrm{ms}$, thus not affecting the transmission of CPMs, as this corresponds to its minimum generation period. On the other hand, the considerably higher traffic density of scenario B and the larger CPMs message sizes cause significantly higher strains to the communication channel, ranging from ~50% CBR (ETSI DCC state “active 3”) up to ~75% CBR (ETSI DCC state “restrictive”), with an average ~65% CBR (ETSI DCC state “restrictive”). Due to the higher message duration $\bar{T_{on}}>0.5\;\mathrm{ms}$ the channel access is more restricted, translating into minimum idle times $T_{off}\geq 1000\;\mathrm{ms}$ for CBRs higher than 60%. Nevertheless, it should be remembered that the levels are depicted only to show when the DCC states would come into effect and where deviations from the DCC activated states occur. As it can be seen, DCC does not play a role also in the high scenario B up to a V2X equipment rate of 25%. Thus, if activated, the reactive DCC would only throttle the CPM transmission for V2X penetration rates higher than 25% in scenario B. 

### 5.3. Environmental Perception

Having analyzed the extent to which the communication channel is strained by the collective perception service, we now investigate its benefit in terms of enhanced environmental perception.

#### 5.3.1. Environmental Awareness Ratio (EAR)

The most extensively investigated environmental perception metric is the EAR. As described in Section 4.2.1, the EAR typically refers to the share of objects known to a station. It may as well represent the fraction, e.g., of known space occupation in a specific region of interest. For the purposes of this investigation, we stick to the former, the Object Awareness Ratio. 

**Figure 10:**
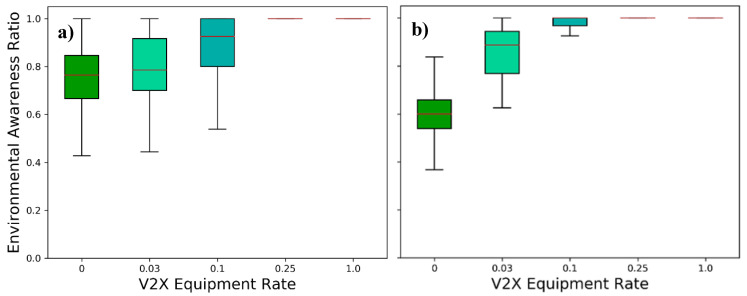
Environmental Awareness Ratio in dependence of the V2X equipment rate for (**a**) the low-density scenario and (**b**) the high-density scenario for an area with a diameter of 250 m, showing typical data range (whiskers), interquartile range (boxes) and median (red-colored line).

The benefit of collective perception in terms of increased environmental perception becomes clear by having a look at Figure 10. While the on-board sensors’ perception is limited in range as can be seen in Figure 10a, where only sporadic occlusion of the sensors’ field of views occurs, the occlusion becomes a major hurdle in denser traffic scenarios [42]. The median EAR for the sensor-only scenario drops from 78% in Figure 10a to 60% in Figure 10b, effectively doubling the proportion of untracked vehicles compared to scenario A. Higher V2X equipment rates, however, lead to an increasing environmental awareness of the traffic participants in both scenarios. Equally expectable is the fact that full market penetrations allow for a perfect environmental awareness within the defined region of interest. Even though some packets in scenario B may be lost due to the high channel load addressed in the previous subsection, the track manager of the environmental model of the vehicles maintains the tracked objects for a certain time by predicting their new states, until the accuracy threshold is reached (see Section 5.1.2). This makes the environmental perception more robust to packet losses as, e.g., compared to [6].

However, when comparing the evolution of the EAR in both scenarios, the higher traffic density presents a much earlier benefit from rising V2X rates as compared to the low traffic scenario. This can be explained by the number of cooperative vehicles within the communication range (see traffic densities in Figure 5). While a 3% market share translates into an average of less than one collaboration partner in the low-density scenario, it corresponds to almost four in scenario B. Thus, an important conclusion is that the performance of collective perception in terms of EAR depends less on the fraction of equipped stations, as it would be the case of cooperative awareness, than on their number.

#### 5.3.2. Detected Object Redundancy (DOR)

**Figure 11:**
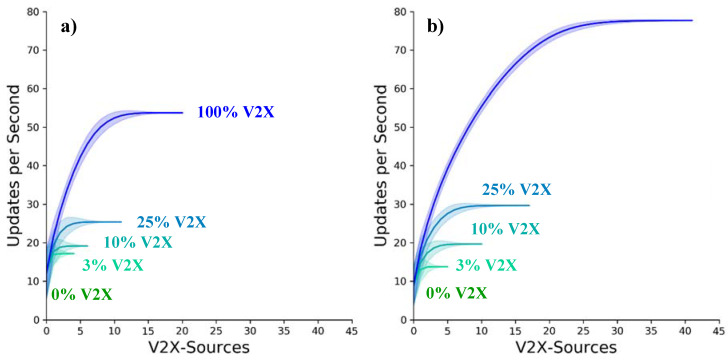
Updates per second for (**a**) the low-density scenario and (**b**) the high-density scenario, displaying the contribution of each connected station in dependence of the V2X equipment rate.

The second investigated metric describing the benefit of collective perception is the DOR introduced in Section 4.2.2. Figure 11a,b show the average received updates per second per object for objects within the area of relevance. The sources from which the data are received are arranged on the respective x-axis according to their contribution. The lines in the figure represent the means and the shaded areas the standard deviation for the different investigated equipment rates. For example, in scenario B the ego vehicle would receive an average of 10 updates per second from its own on-board sensors. The best reporting V2X source would provide another 2–5 updates depending on the V2X equipment rate. As the sources are sorted in terms of their contribution, the latter gradually decreases to an almost negligible minimum for the less reliable cooperation partners.

As expected, the detection redundancy can be considerably enhanced by collective perception in both scenarios. While some of the cooperating stations are close enough to the object and may include it at high rates, those further away are limited by the reduced visibility of the object due to the reach of the sensor system and the shadowing caused by other traffic participants. A second effect reducing the number of updates by a cooperating station is the quality of the communication. It is responsible for a decreasing number of received updates with increasing distance between transmitting and receiving station. For this reason, the bulk of the updates come from 10 and 20 sources in scenario A and B respectively, and all further sources only contribute sporadic measurements.

The two main advantages of the increased DOR are: (i) the higher update frequency and thus lower age of information, and (ii) the higher integrity and higher accuracy of the fused object data due to an increased number of independent sources. While the former may be increased from 13 over 17, 19 and 25 to 53 Hz for equipment rates of 0, 3, 10, 25 and 100% in the low-density scenario, the increase is again more pronounced for the high-density scenario, going from 10 over 13, 19, 29 to 76 Hz for the same equipment rates. It is noteworthy that the variability of the update rates in the low-density scenario is considerably higher, due to the lower number of vehicles involved. Further, it should be noted that the main V2X source is typically, but not always, the transmitting vehicle itself, sharing information concerning its own state over the transmitting station container of the CPM. An example is vehicle 807 in Figure 7, generating CPMs with 10 Hz. The main responsible for this high generation frequency is vehicle 808, driving at almost 200 km/h. The latter, however, would broadcast the state of 807 at a lower frequency due to its lower speed. On the other hand, vehicle 807 would also be the most relevant source for vehicle 808 for the same reasons, presenting the somewhat rarer case when the main source is not the transmitting station itself. The 100% self-inclusion rate is also the reason for the unproportioned increase of the update period for the 3% equipment rate with respect to the higher rates.

The second advantage is the increased reliability of the managed data due to a higher number of sources. It was found that while for the low-density scenario with a 3% V2X market share the average number of sources is still lower than one, it quickly increases to over eight for the full market penetration. With an average of 2.5 additional V2X sources for each object in the area of relevance, the 25% equipment rate can already ensure some level of trust. In scenario B this point is reached with only 10% equipment rate, proving the statement, that collective perception depends more on the number of collaboration partners than on their relative number.

#### 5.3.3. Object Tracking Accuracy (OTA)

One of the two benefits of the DOR analyzed in the previous sub-subsection was the higher update rate for tracked objects. While fresh measurements contribute to enhance the precision of the GEM, highly outdated data lead to a quick degradation of the latter. For this reason, the most important benefit of a high update rate is the higher tracking accuracy of the objects in the environment of the station. 

**Figure 12:**
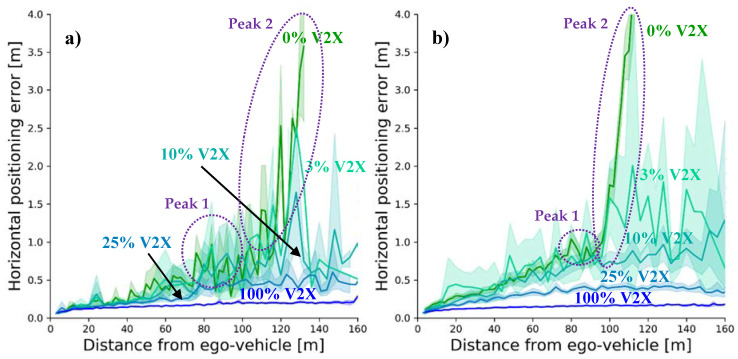
Object Tracking Accuracy and their standard deviations for (**a**) the low traffic scenario and (**b**) the high traffic scenario in dependence of the V2X equipment rate and the distance to the ego vehicle.

Figure 12 shows the horizontal portion of the object tracking accuracy as a function of the object’s distance from the ego vehicle. The figure shows a rapid deterioration of the OTA for a vehicle only relying on its own sensor system. This is especially true for the two peaks located at 80 m and somewhat more than 100 m distance from the ego vehicle. While the former is generated by new objects entering the field of view of the sensor system, the latter corresponds to the objects leaving it. In the time dimension, the trajectory of an object starts at Peak 1 when it is detected for the first time, from there it approaches the origin on the x axis until it passes the ego vehicle and then moves away again before it finally leaves the LEM at Peak 2. Both peaks are blurred due to the objects’ relative speed. When a vehicle is first detected, it is generally approaching the ego vehicle. In case this approach is slow, the tracking error will decrease already at far distances. On the other hand, vehicles approaching at high speeds may enter deeply into the ego vehicle’s perception range before the necessary number of measurements has been gathered for a good convergence of the OTA. The contrary is true for an object leaving the sensors’ field of view. The lower its speed, the longer it will take it to drive away from the ego vehicle, leading to a higher deterioration of the OTA due to system noise. While the second peak is clearly visible for the 0% equipment rate, the first peak is attenuated as a result of merged OTAs from vehicles entering the ego’s field of view and those leaving it.

Despite the amount of displayed data, the benefit of collective perception is clearly visible: (i) it enhances the positioning accuracy within the vehicle sensors’ field of view, and (ii) it extends the perception range. While the significantly high performance of collective perception with equipment rates of 25 and 100% is immediately apparent, this effect is much less visible for lower V2X market shares. With 3% it even seems to perform worse at first sight. This can however be explained by the additionally introduced measurement errors coming from the GNSS positioning accuracy. The received V2X data relative to the transmitting station need to be transformed into absolute coordinates. In a second step, the data then have to be transformed back into coordinates relative to the receiving station to be merged into its global environmental model. Thus, the absolute positioning errors of both connected stations are added to the measurement errors of the detection. For this reason, object measurements obtained from collective perception with a certain sensor will be of a poorer quality than their peers obtained from an identical sensor on the ego vehicle. It is important to notice, however, that despite its poorer quality, V2X gathered data still enhance the environmental perception in all cases. The effect that seems to deteriorate the OTA at low equipment rates deals from the higher environmental perception of the vehicles. While the OTA of objects already tracked by the ego-sensors can be enhanced by further V2X measurements, objects not yet tracked by them will be newly incorporated into the GEM. As the only measurements of these objects come from V2X sources, their OTA will be worse than that of objects only tracked by the ego-sensors. In other words, Figure 12 averages over already tracked objects with V2X enhanced OTAs and V2X-only tracked objects, resulting in the erroneous impression that collective perception may decrease the OTA of a tracked object. However, as explained, this is obviously not the case.

**Figure 13:**
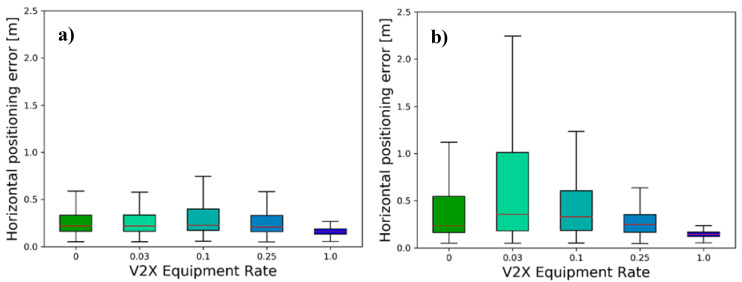
Object Tracking Accuracy for (**a**) the low traffic scenario and (**b**) the high traffic scenario in dependence of the V2X equipment rate aggregated for the objects within the security range, showing typical data range (whiskers), interquartile range (boxes) and median (red-colored line).

Further aggregation of the data with respect to the distance between the object and the ego vehicle leads to the box plots represented in Figure 13. They offer another perspective of the tracking accuracy in dependence of the V2X equipment rate. As it can be seen, the OTA first increases in both scenarios with increasing market share, before it falls to much lower values. This increase of the OTA has its origin precisely in the effect described before. More objects can be included into the GEM (compare EAR in Section 5.3.1), however, they possess below average OTA values, increasing the average positioning error. With increasing numbers of updates coming from additional collaboration partners, the average positioning error can be reduced, finally falling under the non-collaborative scenario despite the much higher EAR. The reason for a later convergence of the low-density scenario is again the lower number of collaboration partners, resulting in a slower increase of the EAR (Figure 10) and thus translating into a lower weight of the additionally tracked objects.

### 5.4. Safety Metrics

The last subsection showed that none of the investigated metrics is sufficient to describe the benefit of collective perception in a stand-alone fashion. The two metrics investigated in this subsection aim at merging the EAR, DOR and OTA into a single target metric.

#### 5.4.1. Environmental Risk Awareness (ERA)

**Figure 14:**
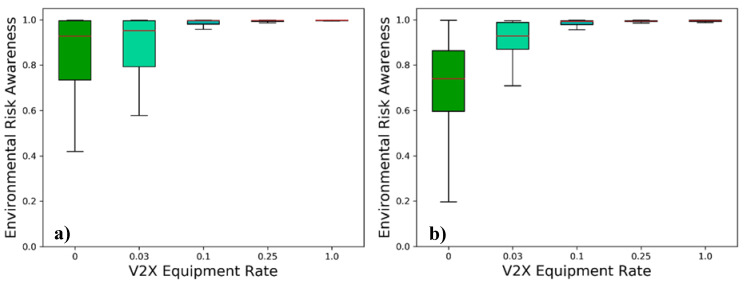
Environmental Risk Awareness for (**a**) the low-density scenario and (**b**) the high-density scenario, showing typical data range (whiskers), interquartile range (boxes) and median (red-colored line).

The ERA introduced in Section 4.3.1 represents the extent to which the risk of the surrounding traffic situation is assessed by a traffic participant within a certain confidence level. Figure 14 shows the ERA for a 95% confidence range in both investigated scenarios, corresponding to the confidence interval specified in ETSI’s TR for collective perception [2]. 

In the stand-alone scenario the vehicles estimate the risk in average 10% lower than it is. It can further be observed that due to the lower sensor shadowing, the risk is more accurately determined in scenario A despite the higher vehicle speeds. As expected, the collective perception-enhanced scenarios outperform the stand-alone benchmark even at low V2X penetration rates. The ERA profits stronger from low V2X equipment rates with higher vehicle densities. As the ERA indirectly depends on EAR, DOR and OTA, this finding is not surprising. With a full V2X market penetration, the risk awareness of the vehicles is almost perfect. These results strongly encourage the deployment of collective perception in future autonomous vehicles, as a highly accurate risk assessment is a strict requirement for their market introduction. However, it is worth reminding that these results correspond to a 1-covariance confidence range, following the specification of the CPM in the TR [2]. For autonomous vehicles, a higher protection level will generally be of interest. Further, the ERA expresses the risk awareness of a traffic participant and is not a direct measure of the vehicle safety (treated in the following sub-subsection). The difference is particularly evident in situations where a reaction is no longer possible. The vehicle may have a perfect ERA; however, its safety is overly critical. For most cases, however, a higher ERA implies a better basis for decision-making, having positive effects on traffic safety, efficiency, and comfort.

#### 5.4.2. Safety Metric

**Figure 15:**
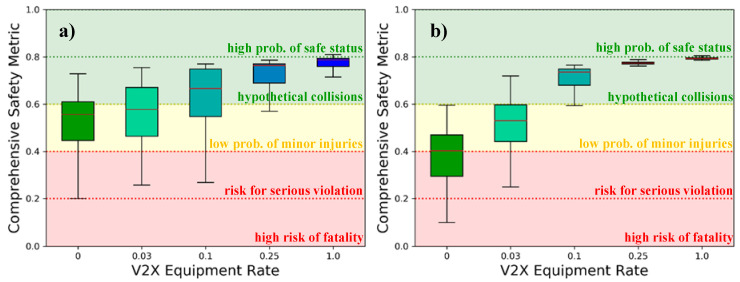
Comprehensive Safety Metric for (**a**) the low-density scenario and (**b**) the high-density scenario with the corresponding safety levels as stated in [44].

Finally, both scenarios are investigated in terms of the provided safety. Figure 15 shows the safety resulting from the corresponding environmental perception of the involved traffic participants. In contrast to the complex computation, the safety value classification provided by [44] allows for a simple interpretation of the results. 

According to the classification, the safety in the stand-alone case can be labeled as “bad” (existing risk for serious violation) in roughly 25%, “good” (low probability of minor injuries) in roughly another 50% and “very good” (hypothetical collisions without minor injuries) in the remaining 25% of the cases in scenario A. Only in rare cases it can be called “insufficient” (high risk of fatality) or “excellent” (high probability of safe status). In scenario B, the effect of the lower speed of the vehicles is outweighed by the poorer environmental perception due to the occlusion of the sensors’ field of view and the lower inter-vehicle distances. This leads to almost half of the results being either “bad” or “insufficient”, thus presenting serious risks to the driver and his environment. The considered sensor equipment would hence be insufficient for a fully autonomous vehicle. While the safety in the high-density scenario increases rapidly with the V2X market share, the development is considerably slower for the low-density scenario, due to effects already discussed. A “very good” safety, with low risk of collisions without fatalities in both scenarios becomes only possible at V2X equipment rates higher than 25%. These findings are remarkable, considering the simplicity of the deployed sensor systems. 

## 6. Conclusions

In this paper, collective perception was examined regarding different performance metrics. To this end, two highway segments with different traffic densities were investigated with the help of TEPLITS, a test and simulation environment for intelligent transportation systems developed in the scope of the public funded project IMAGinE. Real vehicle traces from the highD dataset recorded with drones on German highways were used to ensure the significance and validity of the results. The vehicles were equipped with non-redundant 360° sensor systems of 85 m range, GNSS receivers and V2X communication according to ITS-G5. The collective perception performance was analyzed based on three categories of performance metrics: (i) effect on the communication channel, (ii) environmental perception, and (iii) safety metrics. In agreement with other studies, collective perception is shown to impact the load of the communication channel, emphasizing the need for appropriate congestion control mechanisms, such as the redundancy mitigation techniques mentioned in ETSI’s technical report of collective perception [2]. Further, collective perception considerably enhances the vehicles’ environmental perception, especially in terms of object awareness, detected object redundancy, and object tracking accuracy. In contrast to cooperative awareness, the performance of collective perception depends primarily on the number of connected stations, rather than on the V2X equipment rate. A new metric was introduced to quantify the extent to which a station can correctly assess the risk of the current traffic situation. It was found that collective perception greatly enhances the risk awareness of the connected vehicles in both scenarios, having positive effects on traffic safety, efficiency, and comfort. Finally, an existing safety metric was adapted to suit its application to collective perception. The simulation results showed that the safety of a vehicle equipped only with the mentioned sensor system is exposed to serious risk of accidents, especially in high traffic density scenarios. As hinted by the previous results, traffic safety considerably increased with collective perception. A result with far-reaching consequences is that only with an equipment rate >25% does the accident risk drop to a level that is acceptable for the deployment of autonomous vehicles equipped with comparable sensor systems.

In view of these findings and the introduced performance metrics, further research directions arise. Regarding the load on the communication channel generated by collective perception, several congestion control techniques have been proposed recently. The effect of the reduction of transmitted data on the service has been analyzed based on the state-of-the-art metrics mentioned in this work, however, the implications on the traffic safety have not been studied thus far. Further, the increased safety provided by collective perception could be investigated in different scenarios, such as intersections and roundabouts, with more sophisticated sensor systems and even a comparison of the two competing V2X technologies ITS-G5 and side link C-V2X in terms of safety would be conceivable.

## Figures and Tables

**Table 1:** Investigated highway scenarios with low (A) and high (B) traffic density.

Scenario	A	B
Speed limit [km/h]	-	120
Total distance driven [m]	342,235	1,120,346
Total time driven [s]	10,298	80,676
Number of vehicles (trucks)	856 (168)	2850 (389)
Number of lanes	3↓↑3	3↓↑3

## Data Availability

This research was carried out based on the highD dataset provided by RWTH Aachen University and fka GmbH. The dataset is free for non-commercial use only and can be obtained from https://www.highd-dataset.com/.
